# A primary health-care intervention on pre- and postnatal risk factor behavior to prevent childhood allergy. The Prevention of Allergy among Children in Trondheim (PACT) study

**DOI:** 10.1186/1471-2458-10-443

**Published:** 2010-07-28

**Authors:** Ola Storrø, Torbjørn Øien, Christian K Dotterud, Jon A Jenssen, Roar Johnsen

**Affiliations:** 1 Department of Public Health and General Practice, Faculty of Medicine, Norwegian University of Science and Technology, N-7489 Trondheim, Norway

## Abstract

**Background:**

This study aimed to evaluate the impact of a primary prevention intervention program on risk behavior for allergic diseases among children up to 2 years of age. The setting was in ordinary pre- and postnatal primary health care in Trondheim, Norway.

**Methods:**

The Prevention of Allergy among Children in Trondheim, Norway (PACT) study invited all pregnant women and parents to children up to 2 years of age in the community to participate in a non-randomized, controlled, multiple life-style intervention study. Interventional topics was increased dietary intake of cod liver oil and oily fish for women during pregnancy and for infants during the first 2 years of life, reduced parental smoking and reduced indoor dampness. A control cohort was established prior to the intervention cohort with "follow up as usual". Questionnaires were completed in pregnancy, 6 weeks after birth and at 1 and 2 years of age. Trends in exposure and behavior are described.

**Results:**

Intake of oily fish and cod liver oil increased statistically significantly among women and infants in the intervention cohort compared to the control cohort. There was a low postnatal smoking prevalence in both cohorts, with a trend towards a decreasing smoking prevalence in the control cohort. There was no change in indoor dampness or in behavior related to non- intervened life-style factors.

**Conclusions:**

The dietary intervention seemed to be successful. The observed reduced smoking behavior could not be attributed to the intervention program, and the latter had no effect on indoor dampness.

**Trial registrations:**

(Current Controlled Trials registration number: ISRCTN28090297)

## Background

A parliamentary bill was presented in 1994 to initiate preventive measures against the rising incidence of asthma, allergy and eczema among Norwegian children during the last decades. In 1997 Trondheim was chosen to develop, implement and evaluate relevant prophylactic measures in collaboration between the political and medical authorities in the community and the Norwegian University of Science and Technology (NTNU). Such interventions should be transferable to other communities for implementation in primary health care without extra cost or time-expenditure. Based on existing evidence and demands in literature the following interventional aims were defined: 1: Reduced parental smoking and environmental second hand smoke (SHS). 2: Increased dietary n-3 polyunsaturated fatty acid (n3-PUFA) intake and fish consumption for women during pregnancy and for their offspring up to 2 years of age. 3: Reduced indoor dampness in homes.

The smoking intervention was chosen as a generally accepted and avoidable risk factor, recognizing the harmful effects of tobacco smoke exposure to pregnant women, mothers and small children [1-6].

When the dietary intervention with n3-PUFA was planned there was conflicting results regarding fatty fish/n3-PUFA interventions in prevention of allergic disease [7-9]. Perinatal maternal dietary supplementation with n3-PUFA was assumed to affect T-cells and antigen presenting cells of the neonates due to altered eicosanoid metabolism[10] and thereby modifying neonatal cytokine levels. If so, supplementation of the maternal diet in pregnancy with n3-PUFA could prevent the development of allergic diseases[11]. Furthermore, dietary n3-PUFA in infancy are positively associated with IgA and sCD14 levels, suggesting a relationship between fatty acid status and mucosal immune function [12].

The clinical effect of n3-PUFA however, was doubtful and larger prospective studies were in demand[13]. In two more recent studies and reviews a regular fish consumption before age 1 appeared to be associated with a reduced risk of allergic disease and sensitization to food and inhalant allergens during the first 4 years of life, and particularly in the offspring of mothers without atopic disease[14,15].

The indoor dampness intervention was based on a Nordic interdisciplinary review of the scientific evidence on associations between exposure to dampness in buildings and health effects (NORDDAMP), together with several other publications at that time[16-19]. There is a connection between damp housing and sensitisation to dust mites and/or moulds and childhood respiratory symptoms [17]. Even exposure to low levels of mite allergen is found to be a significant risk factor for sensitization [20]. Later results indicate that building dampness and mould are associated with approximately 30-50% increases in a variety of respiratory and asthma-related health outcomes [21,22].

An applicable and structured interventional program for simultaneous intervention on all three interventional topics was then developed.

A primary assignment was to find out to what extent pregnant women and parents would comply with the behavior recommendations in the intervention program when implemented in a real-life setting. Thus the study aimed to evaluate the impact of a primary prevention intervention program on risk behavior for allergic diseases among children in a pre- and postnatal primary healthcare setting in Trondheim, Norway.

## Methods

### Study population

In 2000 the PACT study was initiated as a cohort study in primary health care in Trondheim, Norway, a community of 165 000 inhabitants and approximately 2100 deliveries per year. Prophylactic measures to induce behavioral changes regarding tobacco smoking, consumption of cod liver oil, oily fish, and indoor dampness were developed in collaboration between general practitioners (GPs), midwives, public health visitors and parents using a Delphi technique[23]. In all, 32 of 35 general practices (altogether 104 GPs), all seven community based midwifes and all 20 maternity health centers in Trondheim agreed to participate. Admission to a control cohort to monitor changes and trends in lifestyle and diet habits and trends in incidence of allergic diseases started in September 2000. These women had a "follow up as usual". All women who received an invitation and gave written informed consent to participate were included in the study with no further selection criteria. Yearly cohorts of pregnant women, children at 6 weeks, 1 year and 2 years of age were recruited consecutively until recruitment to the intervention started for the actual year-group, and the last 2 year old was included in December 2004. Information on risk factors and life-style were collected in parental self-reported questionnaires in ordinary consultations during pregnancy, 6 weeks after birth and at scheduled check-ups at 1 and 2 years of age. End points such as allergic disease together with a health inventory were completed in separate questionnaires at 2 and 6 years of age[24].

Recruitment to the intervention cohort started in June 2002, with all participants included by GPs and midwifes during pregnancy. Inclusion criteria were as for the control cohort, with a follow up and data collection with the same questionnaires at the same ages as in the control cohort. This inclusion ended in June 2006. The collection of questionnaires at 2 years after birth was completed in March 2009. The data collection will continue until the children are 6 years old, providing cross-sectional data in both cohorts permitting estimates for trends in exposure, behavior and disease.

### Interventional topics and strategies

All interventions were initiated at first scheduled consultation in pregnancy as soon as the informed consent form was signed.

In Norway a daily supplement of cod-liver oil is very common and already recommended for children and adults alike. In the intervention program we aimed for a dietary intake of n3-PUFAs of at least two meals of oily fish a week and 5 ml cod-liver oil a day during pregnancy (5 ml cod liver oil = 1.2 g N-3 PUFA). Cod liver oil was to be introduced to the child from 4-6 weeks of age increasing to 5 ml/day, and oily fish at least twice a week from 6 months of age as dinner or sandwich spread. We did not intervene on intake of vegetables, breastfeeding, formula or other dietary factors.

In the smoking cessation and SHS intervention the group adapted a clinic-based brief "5A" office intervention based on the "A Clinical Practice Guideline for Treating Tobacco Use and Dependence"[25,26].

The indoor dampness interventional strategy provided advice on how to detect and advice on how to reduce indoor dampness and its consequences. Simple advice regarding inspection of signs of dampness as damage due to moisture on walls and floors, mould and/or musty smell was given. Solutions such as simple ventilation by opening windows regularly and avoiding drying of clothes in living rooms were recommended.

### Implementation

In Norway the normal schedule at the time constituted of 8-10 prenatal consultations with a GP or midwife from week 8-10 in pregnancy followed by 10 postnatal consultations with public health visitors at maternity care centers during the child's first year of life. Thereafter the schedule was to see all children at 15 and 24 months. Children regarded at risk for disease were seen more often. The intervention program was implemented as the recommended maternity care life-style counseling program throughout the city, regardless of participation in the PACT-study or not. The officially recommended time-schedule for primary care pre- and postnatal follow-ups in Norway was followed for both cohorts. This program was accessible and recommended for all women, free of charge, and with a nation-wide attendance rate of nearly 100% in both urban and rural areas. The interventions were repeated at scheduled consultations throughout pregnancy until 2 years postnatal, either simultaneously or sequentially, at least five times for each topic both pre- and postnatal, assuming no extra time expenditure. All participating GPs, midwives and nurses were offered a course on the interventional program and strategies, including a three hours course on smoking cessation and relapse prevention to ensure a consistent intervention and improve on possible low self-confidence in life style counseling skills [27]. Written guidelines, including self-help material, were distributed to all primary care health professionals and the intervention was designed to be the best of one's ability be delivered as an integrated part of ordinary maternity care in a personalized and individually adapted way, based on possible former knowledge of the family in question. The intention was to obtain awareness, agreement, adoption and adherence to the interventional topics for both health professionals and recipients. Interference with the health professionals and participants from the study-group was limited to what might have been expected from officials in ordinary clinical practice. In accordance with this there was no monitoring of the implementation of the intervention program activity among the health professionals in the intervention group.

## Questionnaires

### Exposure variables

Validated questionnaires for the actual age-group were not available at the time, so questions were adapted from various sources [16,28-32]. Information regarding age for introduction of a variety of food products, including different kinds of porridge, bread, vegetables, fruit, commercially produced baby dinner, homemade baby dinner, fish, cows' milk, and eggs were obtained when the children were 1 year of age. Duration of breastfeeding, time for introduction and type of infant formula, vitamins and cod liver oil, information on housing conditions, parental smoking at start of pregnancy and 1 year after birth, indoor smoking, and pregnancy related complications were collected. Information on consumption of vegetables, cod liver oil, lean fish (cod and coalfish) and oily fish (redfish, halibut, salmon, trout, herring and mackerel) as dinner and sandwich spread were collected by using validated semi quantitative food frequency questions with six categories: never, less than once a week, once a week, twice a week, three times a week and four times a week or more, and re-categorized later in the analyses[33,34].

Parental smoking during pregnancy was assessed with two questions where the women were asked if they or their partner were smoking at start of pregnancy, if they were smoking now and daily and/or weekly cigarette consumption. A separate question was asked about the total numbers of cigarettes smoked indoors. Smoking was coded as a dichotomous variable, if subjects were smoking more than one cigarette a week they were coded as smokers, if the answer was no they were coded as non-smokers, and if the answers to all questions on smoking were missing they were coded as missing.

Housing conditions and indoor dampness were assessed with one question asking for eight different indicators on indoor dampness, as mould or musty smell, moist cardboard and newspapers after storage, dew on windows, moist spots on ceilings, walls or wallpapers, leakage detection on water pipes or faucet, leakage from roof or ground, or moisture in floors. Dampness index was defined by the sum of reported dampness indicators with a sum ≥3 as cutoff.

### Outcome variables

The questionnaire on health was completed when the child was 2 and 6 years old and adapted to our age group from ISAAC [35]. This questionnaire was tested for reliability in a separate study [24]. No biomarkers regarding nicotine, fatty acids or indoor dampness were used.

### Non-participant study

To investigate if there was a selection bias in the PACT-study we conducted an additional non-participants-study where 391 parents who consecutively visited different maternal postnatal care centers were included, 172 were participants in the PACT-study and 219 were non-participants. They were asked to complete a short and anonymous questionnaire on age, socioeconomics, allergic disease and smoking behavior.

## Approvals

The Regional Committee for Medical Research Ethics for Central Norway approved of the study (Ref 120-2000). The study was granted a license by the Norwegian Data Inspectorate to process personal health data and one of the parents signed a written informed consent form (Ref 2003/953-3 KBE/-).

## Statistical analysis

Reporting and analyses are presented according to recommendations in TREND [36]. To estimate a change in exposure before and after implementing the intervention program, parental reports on behavior both intervened and not intervened upon were compared. Population size in the intervention cohort was based on a prevalence of asthma among 6 years old children estimated to 5%. To identify a reduction in prevalence of 40% or more, the population size had to be 2100 children (Alfa 0.05 and a power of 90%). With an expected 30% lost to follow up we needed to include 3000 children in the intervention cohort.

**Figure 1:**
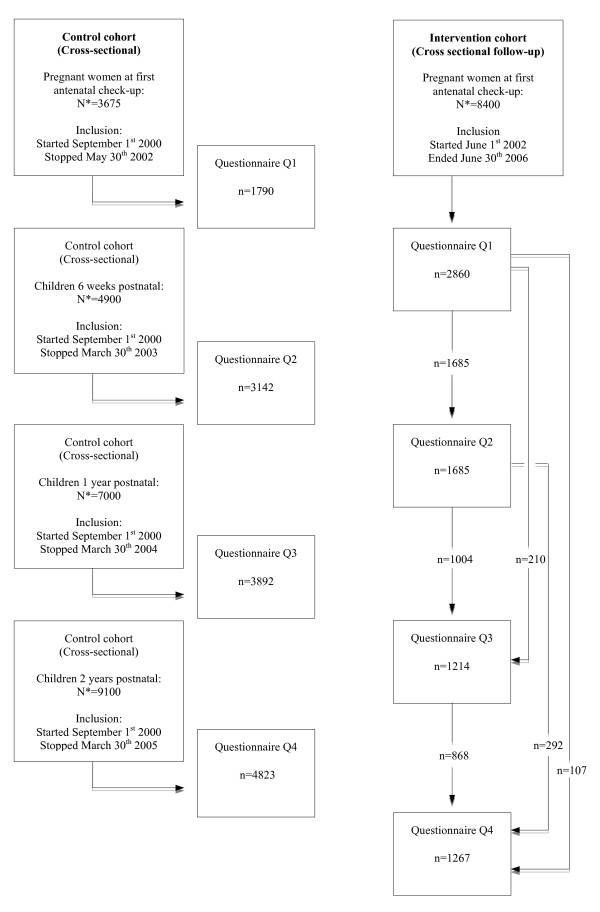
**Flow-chart for the PACT-study 2000 - 2009**. A total of 7845 participants in the control cohort completed 13647 self-reported questionnaires from 2002-2005. The intervention cohort was completed in March 2009. The arrows to the right in the intervention cohort, shows when and how many participants completed the questionnaires at each scheduled follow up. * Total population of children born in Trondheim during inclusion period. Q1 = Questionnaire on behavior and risk factors at first antenatal check-up during pregnancy. Q2 = Questionnaire on behavior and risk factors at 6 weeks of age. Q3 = Questionnaire on behavior and risk factors at 1 year of age. Q4 = Questionnaire on behavior and risk factors at 2 years of age. In this paper Q2, Q3 and Q4 in both cohorts have been used.

SPSS for Windows^® ^ver.15.0 (Chicago, Ill. USA) was used for all statistical analyses. Cross- sectional data were collected for annual cohorts using questionnaires Q2, Q3 and Q4 (Figure 1).

With binary logistic regression models prevalence of exposure factors throughout the study period was used to estimate p for trend in both cohorts. Comparisons between the cohorts were tested with the Chi-square statistics for binomial data and independent t-test for continuous data. Confidence interval (CI) was based on binomial distribution for dichotomous data, and normal distribution for continuous data. Level of significance was set to p = 0.05, two-tailed. Comparisons between the cohorts were performed for women during pregnancy and breastfeeding and for children during first and second year of life.

Confounding factors were identified by *a priori *knowledge, and maternal age, parity, parental allergic disease and homeowner status were tested in several models and decided as the resulting set of covariates.

## Results

### Population

There were no significant differences regarding maternal age, gender, and birth weight between the cohorts. In the intervention cohort, there were fewer primiparous and single women, and there were more homeowners (Table 1).

**Table 1:** Characteristics of parents and children participating in the intervention cohort and the control cohort

	**Cohorts**		
	**Intervention**	**Control**	
		
	**Mean (SD)**		
Age mother (years)	29,5 (4,3)	29,2(4,7)	
Education mother (years)	15,7 (2,5)	15,4 (2,6)	
Education father (years)	15,1 (2,9)	15,0 (3,1)	
Birthweight (grams)	3610 (544)	3590 (573)	
	
	**N (%)**		**p-value**
Gender (male)	48,4	50,1	0,16
Primiparous	44,9	52,2	<0,001
Single mother	1,9	4,3	<0,001
Parental atopy*	68,5	69,4	0,56
Homeowner**	84,1	78,5	<0,001

*Mother and/or father ever had asthma, allergic rhinoconjunctivitis or eczema.

** Assessed at 6 weeks postnatal

### Drop-outs from the intervention cohort

When comparing the participants in the intervention cohort to the drop-outs, the age of the mothers were 29.8 (SD: 4.21) and 29.0 (SD: 4.56), the numbers of maternal smokers were 241 (19.9%) and 366 (23.9%), and 1044 (83.5%) and 1135 (71.2) were homeowners, respectively. There were no differences between the participants and drop-outs regarding education, parity, parental atopy, and in keeping pets.

### Behavioral changes

#### The dietary intervention

During pregnancy the intake of cod liver oil more than four times a week increased significantly from 42% to 66% in the intervention group compared to the control group (Table 2). The women also ate both oily fish and lean fish more often during pregnancy in the intervention group compared to the control group, and there were no time trends regarding diet during pregnancy either in the intervention group or in the control group (Table 2). Among the children we found a very high and equal proportion of approximately 60% having cod liver oil supplement at 6 weeks postnatal in both cohorts (Table 2). During the first 2 years of life the proportion of infants continuing the cod liver oil supplement was about 10 percentage points higher in the intervention cohort (Tables 3 and 4). The proportion having oily fish at least once a week was about 14 percentage points higher at 1 year and 2 years of age in the intervention group compared to the control group (Tables 3 and 4), and there was a positive time trend for eating oily fish in both cohorts at 1 and 2 years of age. There was a statistically significant higher intake of lean fish in the intervention cohort at 1 year postnatal, changing to a significantly higher intake in the control cohort at 2 years of age with no time trend in either cohort. The overall fish intake at one year of age was higher in the intervention cohort, but at 2 years of age the difference was no longer statistically significant (Tables 3 and 4).

**Table 2:** Different exposure and risk behaviours assessed at 6 weeks after birth and the hange in annual prevalence for the intervention cohort and for the control cohort (PACT 2009).

	Intervention Cohort (n = 1685)					Control Cohort (n = 3142)				
	**Rate**	**%**	**95% CI**	P trend*	**Rate**	**%**	**95% CI**	P trend*	OR*	**95% CI**

At start pregnancy										

Maternal smoking	283/1636	17.3	15.5-19.2	0.001	702/2969	23.6	22.2-25.2	0.01	0.70	0.60-0.82
Paternal smoking	292/1594	18.3	16.5-20.3	0.001	652/2803	23.3	21.7-24.9	0.03	0.80	0.68-0.94
During pregnancy										

Maternal cod liver oil 4 times a week or more	1098/1660	66.1	63.8-68.4	0.59	1288/3055	42.2	40.4-43.9	0.16	2.44	2.15-2.78
Maternal oily fish intake once a week or more	624/1553	40.2	37.8-42.6	0.61	858/2812	30.5	28.8-32.2	0.36	1.51	1.32-1.72
Maternal lean fish intake once a week or more	658/1666	39.5	37.2-41.9	0.61	807/3055	26.4	24.9-28.0	0.40	1.80	1.58-2.06
Maternal vegetable almost daily	876/1476	59.3	56.8-61.9	0.25	1548/2704	57.2	55.4-59.1	0.15	1.06	0.93-1.21
At 6 weeks after birth										

Maternal smoking	87/1634	5.3	4.3-6.5	0.002	317/2934	10.8	9.7-12.0	<0.001	0.55	0.42-0.70
Paternal smoking	200/1587	12.6	11.1-14.3	0.04	593/2768	21.4	19.9-23.0	0.01	0.59	0.49-0.70
Keeping dog in house	142/1566	9.1	7.7-10.6	0.69	289/2826	10.2	9.16-11.4	0.95	0.83	0.67-1.03
Keeping cat in house	174/1566	11.1	9.7-12.8	0.58	251/2826	8.9	7.9-10.0	0.90	1.32	1.07-1.64
Indoor dampness Index ≥3	69/1672	4.1	3.3-5.2	0.66	129/3062	4.2	3.6-5.0	0.88	1.08	0.79-1.46
Breastfeeding	1644/1665	98.7	98.1-99.2	0.39	3061/3110	98.4	97.9-98.8	0.81	1.00	0.59-1.71
Child having cod liver oil supplement	1011/1665	60.7	58.4-63.0	0.57	1786/3102	57.6	55.8-59.3	0.66	1.03	0.93-1.21

Comparisons of risk behaviour between the intervention cohort and the control cohort are presented as OR with 95% CI (Based on logistic regression). Change in annual prevalence (intervention cohort 2004-2007, control cohort 2000-2003) presented as p- value for trend. *Adjusted for maternal age, parity, parental atopy and homeowner.

**Table 3:** Different exposure and risk behaviours at 1 year after birth and the change in annual prevalence for the intervention cohort and for the control cohort (PACT 2009).

	Intervention Cohort (n = 1214)					Control Cohort (n = 3892)				
	**Rate**	**%**	**95% CI**	P trend*	**Rate**	**%**	**95% CI**	P trend*	OR*	**95% CI**
At 1 year after birth										

Maternal smoking	105/1198	8.8	7.3-10.5	0.17	720/3755	19.2	18.0-20.5	<0.001	0.47	0.38-0.59
Paternal smoking	148/1144	12.9	11.1-15.0	0.15	724/3468	20.9	19.6-22.3	<0.001	0.64	0.52-0.77
Keeping dog in house	95/1139	8.3	6.9-10.1	0.11	318/3560	8.9	8.0-9.9	0.74	0.90	0.71-1.16
Keeping cat in house	122/1139	10.7	9.0-12.7	0.87	312/3560	8.8	7.9-9.7	0.46	1.33	1.06-1.67
Indoor dampness index	47/1205	3.9	2.9-5.2	0.44	158/3800	4.2	3.6-4.8	0.27	1.00	0.72-1.41
Children's diet at 1 year of age										

Exclusively breastfed 4 months or more	957/1206	79.4	77.0-81.5	0.008	274/3856	71.1	69.6-72.5	0.14	1.46	1.25-1.72
Cod liver oil 4 times a week or more	568/1209	47.0	44.2-49.8	0.34	1497/3858	38.8	37.3-40.4	0.77	1.27	1.11-1.46
Any kind of fish once a week or more	712/1213	58.7	55.9-61.4	0.03	1879/3872	48.5	47.0-50.1	0.06	1.53	1.33-1.74
Oily fish once a week or more	445/1213	36.7	34.0-39.4	0.02	908/3878	23.4	22.1-24.8	<0.001	1.88	1.63-2.17
Lean fish once a week or more	582/1204	48.3	45.5-51.2	0.29	1679/3855	43.6	42.0-45.1	0.46	1.24	1.08-1.42
Vegetables almost daily	889/1198	74.2	71.7-76.6	0.76	2910/3814	76.3	75.0-77.7	0.79	0.87	0.74-1.01

Comparisons of risk behaviour between the control cohort and the intervention cohort are presented as OR with 95% CI (Based on logistic regression). Change in annual prevalence (intervention cohort 2005-2008, control cohort 2000-2004) presented as p- value for trend. *Adjusted for maternal age, parity, parental atopy, homeowner.

**Table 4:** Different exposure and risk behaviours at 2 years after birth and the change in annual prevalence for the intervention cohort and for the control cohort (PACT 2009).

	Intervention Cohort (n = 1267)					Control Cohort (n = 4826)				
	**Rate**	**%**	**95% CI**	P trend*	**Rate**	**%**	**95% CI**	P trend*	OR*	**95% CI**
At 2 year after birth										

Maternal smoking	123/1244	9.9	8.4-11.7	0.87	885/4661	19.0	17.9-20.1	<0.001	0.50	0.41-0.61
Paternal smoking	136/1183	11.5	9.8-13.4	0.11	760/4217	18.0	16.9-19.2	<0.001	0.62	0.51-0.75
Keeping dog in house	96/1198	8.0	6.6-9.7	0.53	368/4403	8.4	7.6-9.2	0.23	0.95	0.75-1.21
Keeping cat in house	133/1198	11.1	9.4-13.0	0.41	398/4403	9.0	8.2-9.9	0.71	1.31	1.06-1.62
Indoor dampness index	46/1259	3.7	2.7-4.9	0.10	170/4740	3.6	3.1-4.2	0.11	1.03	0.74-1.45
Children's diet at 2 year of age										

Cod liver oil 4 times a week or more	539/1257	42.9	40.2-45.6	0.43	1609/4767	33.8	32.4-35.1	0.80	1.44	1.27-1.64
Any kind of fish once a week or more	926/1258	73.6	71.1-76.0	0.87	3398/4782	71.1	69.8-72.3	0.96	1.13	0.98-1.30
Oily fish once a week or more	625/1264	49.4	46.7-52.2	0.04	1679/4809	34.9	33.6-36.3	<0.001	1.85	1.63-2.10
Lean fish once a week or more	768/1256	61.1	58.4-63.8	0.61	3086/4776	64.6	63.3-66.0	0.07	0.85	0.75-0.97
Vegetables almost daily	630/1252	50.3	47.6-53.1	0.25	2340/4735	49.4	48.0-50.8	<0.001	1.01	0.89-1.15

Comparisons of risk behaviour between the control cohort and the intervention cohort are presented as OR with 95% CI (Based on logistic regression). Change in annual prevalence (intervention cohort 2006-2009, control cohort 2000-2005) presented as p- value for trend. *Adjusted for maternal age, parity, parental atopy, homeowner.

#### Smoking intervention

A significant and stable decline in maternal and paternal smoking frequencies was reported from start of pregnancy, through 6 weeks postpartum, and at 1 and 2 years postnatal (Tables 2, 3 and 4). The smoking frequency was almost halved at all four assessment points of time in the intervention cohort compared to the control cohort with a minimum for maternal smoking of 5.3% at 6 weeks postnatal and a minimum of 11.5% for paternal smoking at 2 years postnatal. There was a continuous annual trend for reduced parental smoking in the control cohort, but no further annual trend in reduced postnatal parental smoking in the intervention cohort at 1 and 2 years after birth (Tables 2, 3 and 4).

#### Housing dampness intervention

Indoor dampness index ≥3 was reported by approximately 4% of the participants and constant over and within both cohorts at 6 weeks, 1 and 2 years of age (Tables 2, 3 and 4).

#### Change in non-intervened risk factors

While there was a reported frequency of some 8%-10% keeping a dog, stable between the cohorts and over time, the frequency of keeping a cat was some 2-percentage points higher in the intervention cohort at six weeks, at 1 and 2 years (Tables 2, 3 and 4).

The proportion of children who ate vegetables almost daily was inconstantly different between the cohorts. At 1 year, relatively fewer children had vegetables almost daily in the intervention cohort, while at 2 years there was no difference (Tables 3 and4). The frequency of breastfeeding at 6 weeks did not differ between cohorts, while the proportion of mothers that reported at 1 year to have breastfed exclusively for more than 4 months was significantly higher in the intervention cohort (Tables 2 and 3).

#### The non-participants-study

A comparison between participants in the PACT-study and the general population demonstrated only minor and insignificant differences regarding mean age and education. There was a tendency towards reporting more allergic disease and less smoking at start of pregnancy among participants in PACT, but this difference was not statistically significant (Table 5).

**Table 5:** The non-participants study

	Non-participants n = 219				Participants n =172		
	**Median**	**Mean**		**Median**	**Mean**		**p-value**
	
Mothers age	30	30.8		30.5	30.7		0.89
Childs age	3	4.5		4	4.4		0.93
Education mother (years)	15	15.1		16	15.6		0.08
Education father (years)	15	15.1		16	15.3		0.64

	**n**	**%**	**95% CI**	**n**	**%**	**95% CI**	**p-value**
	
Atopy in family	120	55.0	48.4-61.6	109	63.4	56.2-70.6	0.1
Mothers smoking start pregnancy	46	21.0	15.6-26.4	28	16.3	10.8-21.8	0.25
Mothers smoking now	23	10.6	6.5-14.7	16	9.3	5.0-13.6	0.74
Fathers smoking start pregnancy	39	18.6	13.5-23.8	32	18.9	13.1-24.8	1
Fathers smoking now	37	17.5	12.5-22.5	23	13.5	8.4-18.6	0.32

## Discussion

The results showed that the dietary intake of lean and oily fish and cod liver oil was statistically significant higher in the intervention cohort, both for mothers during pregnancy and for children during the first 2 years of life. Parental smoking prevalence was generally low postnatal, particularly among the mothers, with a statistically significant difference between the cohorts. There was, however, a statistically significant annual trend in the control cohort. There was no difference between the cohorts regarding an indoor dampness index ≥3. Pregnancy and the first years of life are a period of frequent contact with health professionals in many countries and a favorable period for implementing relevant life-style interventions. Which health professionals are responsible and what recommended schedules to follow may differ between nations, but primary care health professionals are generally encouraged to inform, educate and promote healthy behavior among pregnant women and parents during infancy. To assess the efficacy of such interventions should be of general interest although the premises may be somewhat different. The PACT study was conducted over an 8 years period with a historical control cohort established over a 2 years period immediately before the intervention started. The intervention was implemented as a guideline by all primary care health professionals to all women in ordinary pre- and postnatal health care, regardless of participation in the study or not. This implied for participants in the intervention cohort that they could return self-reported questionnaires at any scheduled follow-up even if they had failed to do so on a previous occasion. This is indicated with figures and arrows in Figure 1.

The comparisons at different age levels permit presentation of behavioral trends. A behavioral trend in the control cohort or in both cohorts simultaneously implies that a possible difference between the cohorts must be interpreted with caution and other explanations than the intervention program should be considered.

Parents in the intervention group seemed to be more persistent in continuing cod liver oil supplement for their infants. There was an annual trend towards increased oily fish intake among children in both cohorts during first 2 years of life probably reflecting a gradual introduction of fish in the diet for all children. There was, however, a persistent and significant difference in oily fish intake between the cohorts at both 1 and 2 years and a shift towards an increased share of lean fish in the diet in the control cohort during the period. A probable interpretation of this may be that oily fish was substituted for lean fish in the children's diet in the intervention group, which was in accordance with the intervention program.

The low smoking prevalence and annual trend towards less smoking in both cohorts during pregnancy are in accordance with earlier findings showing significantly increasing difference in smoking cessation between pregnant women in Trondheim and the comparable city of Bergen and all of Norway in the actual time period [26]. The PACT study period coincided with new legislation on smoking in public places and ongoing national campaigns against smoking. The increased smoking cessation rate observed among pregnant women in Trondheim compared to Bergen and Norway could possibly be a consequence of the ongoing PACT-project as such, with the increased focus on life-style factors during pregnancy and infancy in general and smoking cessation in particular. Interestingly, the continuous smoking intervention did not seem to have any additional effect on the few remaining smokers in the intervention group.

We observed no difference in the housing dampness index between the cohorts. This may reflect a low adherence to the housing dampness intervention. Indoor climate was an unknown and unaccustomed subject for intervention among both health professionals and recipients. Even more expensive and extensive actions as improved roofing and drainage of buildings could have been recommended, but the program had no resources to follow up on this level. The stable fraction of approximately 4% reporting indoor dampness index ≥3 within and between both cohorts at all ages indicates that the question on this topic was highly reliable.

The effectiveness of the three interventions will be assessed by changes in incidence of allergic disease in an upcoming separate paper. The impacts of the dietary intervention however, can be based on inference from an earlier PACT study on the associations between cod liver oil and oily fish and atopic disease[37]. We found that weekly intake of oily fish at 2 years of age gave an OR for atopic eczema/dermatitis (AD) of 0.57 (95%CI: 0.35-0.94) compared to intake less than weekly. This gives an absolute risk reduction of 7.5% (from 18.2% to 10.7%) at 2 years. With a prevalence of eczema of 15.1%, some 184 of 1213 children would have AD of which 13 cases would be prevented with a 14.5 percent points increase in oily fish consumption, corresponding to a 1.1% reduced prevalence of AD at 2 years of age. It is however, a possibility that a simultaneous intervention on smoking and indoor dampness can give a different outcome.

The strengths of the study are the controlled cohort design with a large number of pregnant women followed prospectively in the intervention cohort, and the assessment of risk-factor behavior that was consistent through the observation period and across cohorts. The non-randomized design was adapted to comply with the assignment to investigate the effectiveness of interventions implemented in the way new guidelines usually are in ordinary primary health care[38]. We decided on a design with a control cohort 1 year in advance of the intervention primarily because a public and community based randomized intervention including the entire primary health care in the municipality would have been impossible to implement without contaminating a co-existing control cohort. Secondly, this design also ensured high conformity between the cohorts regarding population size, race/ethnicity, maternal educational level, income, environment, urbanization and social characteristics [39]. This was supported by the results from the additional non-participants-study that included 391 parents, indicating no major selection bias. Only self reported questionnaires were used, as this is a common and feasible way of assessing information in large epidemiologic studies[32,40]. For smoking behavior self reported questionnaires are known to have equal or better reliability, compared to interviews using a structured questionnaire [41,42].

A potential weakness may be that the cohort design with a 1-year difference between the control cohort and intervention cohort might have biased the results toward a better effectiveness of the intervention because of possible annual trends. Although the intervention program was adopted as the official prophylactic program in the community, and nearly 100% of pregnant women visit their GP regularly, only some 34% of the eligible pregnant women participated in the PACT study. The dropouts at 2 years in the intervention cohort was statistically different from the participants regarding maternal age, maternal smoking at start pregnancy and homeowner. This indicates some selection bias at two years in the intervention cohort to be taken into consideration when evaluating the findings.

The inclusion rate decreased substantially during the inclusion period and it was decided to stop at 2860 included. With time health professional reduced their follow-up intensity resulting in a falling follow-up rate, much greater than anticipated. We have however, reason to believe missing was mainly at random in this study. Almost no participants withdraw from the study in the study period, and the reports we have from health professionals indicate that investigators experienced weariness or forgot to present new questionnaires to the participants at ordinary visits as the main reason for missing. Decreasing awareness in long lasting prospective studies causing low participation has been described earlier[43]. The 2100 needed for analysis was based on power estimates for 40% reduction in prevalence of asthma at 6 years, and do not necessarily apply to the reported differences in behaviour in this paper. We think the differences we have found are reliable. If the differences are small, it is certainly a possibility for type II error, meaning that due to the lack of power we erroneously claim a difference non-significant. On the other hand, it is unlikely that the effect of an intervention is overestimated.

It was a requirement specification in this study to have as close to a real-life approach as possible, meaning that the intervention was implemented in the form of a guideline to the participating health professionals. It was a deliberate choice not to follow up on this intervention more than what is usual for guidelines in general when introduced in primary health care. The implementation strategies were considered modest in accordance with the real-life demand, and the effectiveness of the interventional program was exclusively based on parental self-reported risk-factor behavior questionnaires [44,45].

## Conclusions

GPs, midwives, health visitor nurses and parents in the community were jointly responsible for developing an intervention program that were implemented as an official community strategy in primary health care without extra cost or time expenditure. The dietary intervention to increase the intake of cod liver oil and oily fish in pregnancy and among mothers and children first 2 years was successful. The observed reduced smoking behavior could not be attributed to the intervention, and there was no effect of the intervention on indoor dampness. Investigations to develop strategies for successful interventions in primary health care are still needed.

## Competing interests

The authors declare that they have no competing interests.

## Authors' contributions

OS and TØ participated in the design and coordination of the study and drafted the manuscript. JAJ participated in the design of the study and the questionnaires. CKD contributed to analysis and presentation of data and finalization of the manuscript. RJ, the principal investigator of PACT, conceived the study, and participated in its design and coordination and helped draft the manuscript. All authors read and approved the final manuscript.

## Pre-publication history

The pre-publication history for this paper can be accessed here:

http://www.biomedcentral.com/1471-2458/10/443/prepub
